# Zoonotic Disease Testing Practices in Pediatric Patients with Meningitis and Encephalitis in a Subtropical Region

**DOI:** 10.3390/pathogens11050501

**Published:** 2022-04-22

**Authors:** Timothy A. Erickson, Shannon E. Ronca, Sarah M. Gunter, Eric L. Brown, Rodrigo Hasbun, Kristy O. Murray

**Affiliations:** 1 Department of Pediatrics, Section of Pediatric Tropical Medicine, Center for Human Immunobiology, Baylor College of Medicine and Texas Children’s Hospital, Houston, TX 77030, USA; timothy.erickson@bcm.edu (T.A.E.); sm22@bcm.edu (S.M.G.); 2 School of Public Health, University of Texas Health Science Center, Houston, TX 77030, USA; eric.l.brown@uth.tmc.edu; 3 McGovern Medical School, University of Texas, Houston, TX 77030, USA; rodrigo.hasbun@uth.tmc.edu

**Keywords:** zoonotic diseases, clinical diagnostics, vector-borne diseases, encephalitis, meningitis, pediatric

## Abstract

Emerging vector-borne and zoonotic pathogens can cause neuroinvasive disease in children; utilization of appropriate diagnostic testing can be low, hindering diagnosis and clinical management of these cases. We must understand factors that influence healthcare providers’ decisions to order diagnostic testing. We reviewed medical charts for pediatric meningitis and encephalitis patients (90 days–18 years) between 2010 and 2017 and analyzed variables associated with testing for known neuroinvasive zoonotic pathogens in the southern United States: West Nile virus (WNV), *Bartonella* spp., and *Rickettsia* spp. Among 620 cases of meningitis and encephalitis, ~1/3 (*n* = 209, 34%) were tested for WNV. Fewer cases were tested for *Bartonella* (*n* = 77, 12%) and *Rickettsia* (*n* = 47, 8%). Among those tested, 14 (7%) WNV, 7 (9%) *Bartonella*, and 6 (13%) *Rickettsia* cases were identified. Factors predicting testing were similar between all agents: clinical presentation of encephalitis, focal neurologic symptoms, new onset seizure, and decreased Glasgow Coma Scale on admission. Cases with a history of arthropod contact were more likely to be tested; however, we did not see an increase in testing during the summer season, when vector exposure typically increases. While our test utilization was higher than that reported in other studies, improvement is needed to identify zoonotic causes of neuroinvasive diseases.

## 1. Introduction

Vector-borne and zoonotic pathogens can cause severe neuroinvasive disease in pediatric populations, resulting in meningitis, encephalitis, and even death [1,2,3,4,5]. Appropriate and timely diagnosis is essential to patient management and appropriate treatment, and it can inform public health and disease prevention measures. Three such pathogens known to circulate in the subtropical regions of the southern United States include West Nile virus (WNV), *Bartonella henselae*, and *Rickettsia* species [6,7,8,9]. However, the testing rate for these infections can be low, causing a gap in knowledge about the frequency of infection and true disease burden, especially in children [10,11,12].

WNV is an arbovirus transmitted primarily by *Culex* spp. mosquitos. While infection is typically asymptomatic, 20–25% of infections result in febrile illness, and less than 1% of adults develop neurological manifestations (West Nile neuroinvasive disease: WNND) [13,14,15,16]. The rate of WNND is notably lower in pediatric patients, with one study from Ohio reporting that only 1 out of 4200 (~0.02%) infected children developed WNND [17]. Despite this, WNV may still represent one of the most commonly identified viral causes of encephalitis in pediatric patients in high-disease-burden subtropical regions such as Houston, Texas, where 24% of children with viral encephalitis were diagnosed with WNV [9,12]. Previous studies have found that <50% of adult patients with meningitis and encephalitis are tested for WNV, and testing was less common in pediatric populations (~25%) [10,11].

*Rickettsia* spp. are a family of vector-borne pathogens divided into the typhus group *Rickettsia* (TGR) and spotted fever group *Rickettsia* (SFGR), which are primarily transmitted by fleas and ticks, respectively. Patients typically present with a fever and rash, and infections can be severe, resulting in hospitalization and even death. Neuroinvasive infections are uncommon, but can occur [2,12,18,19,20,21]. The incidence of *Rickettsia* spp. has been increasing rapidly in Texas over the last two decades, and effective delineation of the role of these species in neuroinvasive disease is important [7]. *Bartonella henselae*, the causal agent of cat-scratch disease, is common in the southern United States and has been noted as a cause of encephalitis in around 3% of children in Texas [8,12].

Information regarding factors leading to testing for these pathogens in children with neuroinvasive disease is lacking. The goal of this observational study was to evaluate the rate of testing and factors driving testing for these important zoonotic pathogens in a large pediatric population in Texas.

## 2. Results

We identified 799 cases of meningitis and encephalitis between 1 January 2010 and 31 December 2017, with 620 cases meeting the criteria for inclusion in this study. The majority of patients had no identified etiology (*n* = 328, 53%), followed by viral (*n* = 213, 34%), autoimmune (*n* = 60, 10%), bacterial (*n* = 13, 2%), and other (IVIG) (*n* = 6, 1%) causes. Most patients (*n* = 411, 66%) had meningitis; the remainder were encephalitic. Approximately one-third of all cases (209, 34%) were tested for WNV, of which 175 (84%) were tested using the arboviral panel (WNV, St. Louis, California encephalitis virus, eastern equine encephalitis virus (EEEV), and western equine encephalitis virus (WEEV)). Fewer cases were tested for *Bartonella* (*n* = 77, 12%) and *Rickettsia* (*n* = 47, 8%). Of those tested, the incidences of positive results for each of these diseases, respectively, were 14 (7%), 7 (9%), and 6 (13%).

We compared the 209 total patients tested for WNV to the 411 who were not tested (Table 1) and found that a patient presenting with encephalitis was more likely to be tested for WNV than a meningitis patient (*p* < 0.0001, OR 12.8, 95% CI 8.4–19.5). Patients were also more likely to be tested for WNV if they had new-onset seizure (*p* < 0.0001, OR 5.5, 95% CI 3.7–8.2), a lower GCS score (*p* < 0.0001, OR 7.6, 95% CI 4.7–12.5), or new-onset focal neurologic abnormalities (*p* < 0.0001, OR 4.9, 95% CI 3.2–7.5). Percent lymphocytes (*p* < 0.001), history of recent mosquito bites (*p* < 0.0001, OR 8.2, 95% CI 4.4–15.8), and number of days of reported symptoms prior to admission (*p* < 0.001) were significant predictors of a WNV test. Patients with increasing leukocyte concentrations were less likely to be tested for WNV (*p* < 0.001). Presentation during WNV season was not a significant factor related to testing (*p* = 0.42, OR 1.14, 95% CI 0.81–1.63).

Many of the variables that predicted WNV testing were also associated with the 77 patients tested for *Bartonella* (Table 1). Clinically, confirmed encephalitis (*p* < 0.0001, OR 16.8, 95% CI 8.5–36.1) was the variable with the strongest association with *Bartonella* testing, while seizure (*p* < 0.0001, OR 4.3, 95% CI 2.5–7.2), lower GCS score (*p* < 0.0001, OR 6.3, 95% CI 3.6–10.8), and focal neurologic symptoms (*p* < 0.0001, OR 5.0, 95% CI 2.9–8.4) were also of importance. Percent lymphocytes (*p* < 0.001, OR 1.03, 95% CI 1.02–1.04) and concentration of CSF glucose (*p* < 0.001)were also significantly different between those who were or were not tested. Patients were less likely to be tested for *Bartonella* with increasing leukocyte concentrations (*p* < 0.001). As expected, presentation during vector-borne disease season was not significant (*p* = 0.38, OR 0.81, 95% CI 0.49–1.34).

A similar variable pattern was associated with *Rickettsia* testing (Table 1). Encephalitis (*p* < 0.0001, OR 5.3, 95% CI 2.7–11.0), decreased GCS on admission (*p* < 0.0001, OR 3.92, 95% CI 2.0–7.6), and presence of a rash (*p* < 0.0001, OR 4.5, 95% CI 2.1–9.4) were clinical signs associated with testing. Only concentration of CSF glucose (*p* < 0.001) in laboratory values was significantly associated with testing for *Rickettsia.* Vector exposure was also significant (*p* < 0.0001, OR 51.4, 95% CI 12.3–296.9). Again, seasonality was not significantly associated with testing (*p* = 0.95, OR 1.02, 95% CI 0.54–1.95).

About half (*n* = 99, 45%) of the cases that were tested for at least one zoonotic disease were also tested for another. As can be seen in the table below, it was most common for cases to only be tested for WNV. Singlet testing for the bacterial organisms was relatively uncommon (Table 2).

## 3. Discussion

In this study, we highlight the factors associated with and underutilization of testing for vector-borne and zoonotic pathogens in a subtropical region where these diseases are known to be transmitted. Similar patterns in testing decision making were seen among the three different pathogens focused on in this study, with a history

Our study identified a relatively high proportion of WNV-positive pediatric patients (7%), even though only one-third of the total patient population was tested. Our findings of WNV in the pediatric population are higher than in predominantly adult studies, such as the California Encephalitis Project (1.2%) and a separate study in Houston (4%) [22]. Notably, the prior Houston study conducted a small subset analysis of pediatric cases and identified only a single case of WNND (1/184, 0.5%). This may have been partially the result of increased testing for WNND in 34% of our cases at TCH compared to the 25% testing rate of children in another Houston-based study [23].

We believe that the WNV-positive cases identified in this study likely represent an underestimation of the true burden of disease in our pediatric population, as an estimated seven million WNV infections have occurred in the United States since its introduction in 1999. While the attack rate of WNND in pediatric populations is lower than in older adults, an estimated two million infections occurred in individuals under the age of 15 [15]. A disproportionate number of cases occur in the southern US, where Texas has one of the highest reported rates of WNV infection and WNND in the United States [15]. Increasing age has been demonstrated to be one of the leading factors associated with testing for WNND, and improvements in testing practices in pediatric populations are needed to determine the true disease burden [10,11]. While there is no specific clinical treatment for WNV other than supportive care, identifying these cases is crucial to public health; hence, the inclusion of WNV on the National Notifiable Disease list [24]. Public health reporting of human cases can inform decision making for vector control measures, thereby preventing further cases.

We found that an encephalitis diagnosis was associated with increased testing for all three of the zoonotic diseases that we assessed. This finding was expected; however, the disparity in results of testing was unexpected. WNV testing was more than four times more likely to be positive in tested encephalitis patients than in meningitis patients. While this finding contrasts with previous adult studies, it is possible that this is a pediatric-only phenomenon, since enterovirus-related aseptic meningitis is more common in children [25]. More research is necessary to validate this finding, but this could be important for future diagnostic testing and patient triage.

Interestingly, this correlation was the opposite for *Rickettsia*. Rickettsial testing was more likely to be positive in meningitis cases, although testing was more likely to be conducted in encephalitis patients. Generally, *Rickettsia* spp. are often reported as causes of encephalitis, specifically related to Rocky Mountain spotted fever (causative agent *Rickettsia rickettsii*), although meningitis can occur in cases of both SFGR and TGR infections on an infrequent basis [18,26,27,28]. Both SFGR and TGR are present with confirmed autochthonous transmission in this region of Texas, although exact identification of species-level causes of disease is a matter of debate [29]. With regards to *Bartonella,* most of the tests (86%) were requested for patients with encephalitis, and as expected, positives were only found in this group.

Vector exposure (mosquito bite, flea/tick contact) was among the strongest associations with testing for a given disease related to WNV or *Rickettsia* spp. Unfortunately, no such measure of exposure was collected for cat contact or scratch, so no comparable variable for *Bartonella* was available. Analyzing vector exposure has some limitations, as these variables are self-reported, and it is often not reported in all cases [29]. While it may not be a reliable variable for excluding cases of disease, the observation of arthropod contact may be able to imply the presence of one of these vector-borne agents in the setting of neuroinvasive disease.

*Rickettsia* spp. testing was the only test associated with the presence of a rash. Convention has suggested that *Rickettsia* spp., especially Texas-endemic *R. typhi*, are traditionally associated with a classic triad that includes rash, fever, and headache; however, studies from past decades have suggested that this triad is an unreliable predictor of infection [23,29,30]. All three of these diseases may present with or without rash, and this may not be an important diagnostic criterion for these agents in the context of neuroinvasive disease [7,18,29,30,31,32]. In fact, rash related to WNV infection was seen more often in younger patients, a fact that has been previously observed [6].

There are important limitations to this study that are worth mentioning. Due to the retrospective nature of the study, any errors in the records could lead to potential information bias. Testing for these agents, while more common than in the comparable literature, was still relatively infrequent. Although the absolute numbers of cases identified are low, encephalitis is a condition resulting from many different agents; zoonoses had a median of seven cases/cause (WNV, *Bartonella*, *Rickettsia*) vs. two cases/cause in non-zoonotic encephalitis in the initial study from which these data were drawn [12]. Single-point samples for intracellular disease, while commonly used in clinical practice, do not provide the same diagnostic value as paired acute and convalescent samples. Future diagnostic studies should consider making further use of molecular testing, but this may not be feasible for some hospital systems. Additionally, the Bonferroni correction used to adjust for multiple comparisons and prevent spurious discoveries may be overly conservative [33,34,35].

This study also possessed many strengths. We presented a large cohort of pediatric neuroinvasive disease patients with a substantial amount of data available for analysis. While other studies of neuroinvasive disease have relied on similar retrospective reviews, one notable strength of our study is that we reduced misclassification bias by incorporating a validated case definition for encephalitis and meningitis. Each case that we included met these gold-standard definitions [36]. Another strength of our study was that our hospital uses a more extensive arboviral testing panel when assessing for WNV infection. This allowed us to scrutinize the results and rule out cross-reactivity with other arboviral pathogens, a concern identified previously [11]. Additionally, most large-scale studies of WNND and the factors associated with testing for WNND have focused on largely adult populations, while our study begins to fill the gap in pediatric knowledge. Finally, we are aware of no previous study of this size that has looked at factors associated with *Rickettsia* spp. or *Bartonella* testing in pediatric patients with meningitis and encephalitis.

In conclusion, the testing rates described here are higher than those reported in previous studies. This indicates a movement in the right direction for improving clinical diagnosis and appropriate care of patients. Overall, we hope to highlight the importance of zoonotic disease testing in pediatric patients presenting with neuroinvasive disease.

## 4. Materials and Methods

Texas Children’s Hospital (TCH) is the largest pediatric hospital in the United States, with more than 30,000 patients admitted per year [37]. For this study, patients who presented to TCH between 1 January 2010 and 31 December 2017 were identified using a search of all ICD 9/10 codes associated with meningitis or encephalitis. The case definition of the International Encephalitis Consortium was applied to identified encephalitis cases as previously described [12,38]. This included altered mental status for >24 h and two of the following: fever, new-onset seizure, leukocytic pleocytosis (>5 cells per mL^3^), focal neurologic symptoms, abnormal neuroimaging results, and abnormal EEG. A modified definition of the National Healthcare Safety Network was applied to meningitis cases, defined as: an organism identified from cerebrospinal fluid (CSF) or leukocytic pleocytosis and fever (>38.0 °C), hypothermia (<36.0 °C), apnea, bradycardia, irritability, or meningeal signs [38,39]. Meningitis may occur in conjunction with encephalitis (meningoencephalitis). When a case met both definitions, they were classified for the purposes of this study as an encephalitis case. Patients were excluded if they were <90 days or >18 years of age at the time of admission. Patients were also excluded if they had a positive CSF culture of fungus or bacteria, microscopic identification of amoebae, or a positive PCR test for a parasitic infection. Additionally, patients were excluded if they had a history of craniotomy or current placement of a VP shunt, as these cases are considered healthcare-acquired ventriculitis or meningitis cases and are unlikely to be related to vector or zoonotic transmission [11].

We acquired demographic data, comorbidities, immune status, clinical features on presentation, Glasgow Coma Scale (GCS) abnormalities (dichotomized as normal (GCS = 15) or abnormal (GCS 3–14)) neuroimaging results, laboratory values, seasonality, days of illness prior to hospital admission, and history of recent vector exposure. Comorbidities and immune status were recorded as dichotomous variables. Laboratory measurements included leukocyte, lymphocyte, glucose, and protein levels. Seasonality was defined for vector-borne diseases based on the prior literature as 1 June–31 October each year [11].

Testing orders for WNV, *Bartonella,* and *Rickettsia* spp. were recorded. WNV testing was most frequently ordered as part of a broad arboviral immune assay panel that included tests for IgM and IgG associated with WNV, St. Louis encephalitis virus, California encephalitis virus, EEEV, and WEEV. Testing for *Rickettsia* was most frequently conducted using an immune assay for IgM and IgG for both TGR and SFGR; similar immunologic testing was conducted for *Bartonella* species. Conventionally, cases of intracellular bacterial infection require secondary testing using a convalescent phase sample; however, this testing is infrequently conducted in this geographic area. Since convalescent testing is rare, we were only able to classify positives as probable cases of TGR and SFGR, as was often the case in similar studies [29,40,41,42].

Statistical analyses were conducted using STATA version 14.0 (Stata Corp., College Station, TX, USA). Statistical comparisons of variables employed chi-square testing and included Fisher’s exact test when a cell contained five or fewer observations; Kruskal–Wallis testing was used to compare continuous values. To reflect the application of the Bonferroni correction for multiple comparisons, the statistical significance level was set to *p* < 0.001.

## Figures and Tables

**Table 1:** Factors associated with zoonotic disease testing.

	Arboviral Testing Requested (*n* = 209), No. (%)	Arboviral Testing Not Requested (*n* = 411), No. (%)	*p*-Value	*Bartonella* Testing Requested (*n* = 77), No. (%)	*Bartonell* a Testing Not Requested (*n* = 543), No. (%)	*p*-Value	*Rickettsia* Testing Requested (*n* = 47), No. (%)	*Rickettsia* Testing Not Requested (*n* = 573), No. (%)	*p*-Value
**Demographics**									
Male	122 (58)	222 (54)	*p* = 0.302	46 (60)	298 (55)	*p* = 0.42	28 (60)	316 (55)	*p* = 0.557
Age (in years)									
<1	9 (4)	55 (13)	*p* < 0.001	1 (1)	63 (12)	*p* = 0.002	0	64 (11)	*p* = 0.010
1–5	55 (26)	96 (23)	*p* = 0.417	20 (26)	131 (24)	*p* = 0.724	11 (23)	140 (24)	*p* = 0.875
6–10	67 (32)	124 (30)	*p* = 0.630	21 (27)	170 (31)	*p* = 0.473	10 (21)	181 (32)	*p* = 0.141
11–14	40 (19)	82 (20)	*p* = 0.810	21 (27)	101 (19)	*p* = 0.073	17 (36)	105 (18)	*p* = 0.003
15–18	38 (18)	54 (13)	*p* = 0.095	14 (18)	78 (14)	*p* = 0.378	9 (19)	83 (14)	*p* = 0.387
Race/Ethnicity									
White, Non-Hispanic	71 (34)	153 (37)	*p* = 0.425	31 (40)	193 (36)	*p* = 0.420	22 (47)	202 (35)	*p* = 0.113
White, Hispanic	82 (39)	173 (42)	*p* = 0.494	32 (42)	223 (41)	*p* = 0.935	14 (30)	241 (42)	*p* = 0.100
Black, Non-Hispanic	34 (16)	50 (12)	*p* = 0.158	8 (10)	76 (14)	*p* = 0.387	5 (11)	79 (14)	*p* = 0.662
Other	22 (11)	35 (9)	*p* = 0.413	6 (8)	51 (9)	*p* = 0.649	6 (13)	51 (9)	*p* = 0.378
Comorbidities noted	46 (22)	51 (12)	*p* = 0.002	16 (21)	81 (15)	*p* = 0.185	15 (32)	82 (14)	*p* = 0.001
**Clinical Features**									
Confirmed encephalitis	146 (70)	63 (15)	*p* < 0.001	66 (86)	143 (26)	*p* < 0.001	33 (70)	176 (31)	*p* < 0.001
Vomiting	97 (46)	216 (53)	*p* = 0.148	32 (42)	281 (52)	*p* = 0.094	20 (43)	293 (51)	*p* = 0.258
Seizure	104 (50)	63 (15)	*p* < 0.001	43 (56)	124 (23)	*p* < 0.001	20 (43)	147 (26)	*p* = 0.012
Fever > 38 C	144 (69)	310 (75)	*p* = 0.083	54 (70)	400 (74)	*p* = 0.512	41 (87)	413 (72)	*p* = 0.024
Glasgow Coma Scale score < 15	80 (38)	31 (8)	*p* < 0.001	38 (49)	73 (13)	*p* < 0.001	20 (43)	91 (16)	*p* < 0.001
Rash	24 (11)	39 (9)	*p* = 0.437	8 (10)	55 (10)	*p* = 0.943	14 (30)	49 (9)	*p* < 0.001
**Epidemiologic Factors**									
Illness onset during WNV season	115 (55)	212 (52)	*p* = 0.417	37 (48)	290 (53)	*p* = 0.378	25 (53)	302 (53)	*p* = 0.949
Days ill prior to admission (median, range)	4 (0–30)	2 (0–30)	*p* < 0.001	5 (0–30)	3 (0–30)	*p* = 0.003	5 (0–30)	3 (0–30)	*p* < 0.001
Vector contact noted on admission	52 (25)	16 (4)	*p* < 0.001	NA	NA	NA	10 (21)	3 (1)	*p* < 0.001

**Table 2:** Testing combinations and results.

Test	N	Positive for *Rickettsia*	Positive for *Bartonella*	Positive for WNV
WNV only	123	-	-	5
*Rickettsia* only	7	2	-	-
*Bartonella* only	3	-	0	-
*Rickettsia* and *Bartonella*	3	1	0	-
WNV and *Bartonella*	49	-	6	5
WNV and *Rickettsia*	15	3	-	1
All	22	0	1	3

## Data Availability

These data are not publicly available.
